# Transdifferentiation of pancreatic cells by loss of contact-mediated signaling

**DOI:** 10.1186/1752-0509-7-77

**Published:** 2013-08-13

**Authors:** Walter de Back, Roland Zimm, Lutz Brusch

**Affiliations:** 1 Center for Information Services and High Performance Computing, Technische Universität Dresden, Dresden, 01062, Germany; 2 Institute of Biotechnology, University of Helsinki, PO Box 56, Helsinki FIN-00014, Finland

**Keywords:** Lineage conversion, Intercellular communication, Reprogramming, Pancreas, Acinar cells, Islet cells, Mathematical model, Multicellular systems biology

## Abstract

**Background:**

Replacement of dysfunctional *β*-cells in the islets of Langerhans by transdifferentiation of pancreatic acinar cells has been proposed as a regenerative therapy for diabetes. Adult acinar cells spontaneously revert to a multipotent state upon tissue dissociation *in vitro* and can be stimulated to redifferentiate into *β*-cells. Despite accumulating evidence that contact-mediated signals are involved, the mechanisms regulating acinar-to-islet cell transdifferentiation remain poorly understood.

**Results:**

In this study, we propose that the crosstalk between two contact-mediated signaling mechanisms, lateral inhibition and lateral stabilization, controls cell fate stability and transdifferentiation of pancreatic cells. Analysis of a mathematical model combining gene regulation with contact-mediated signaling reveals the multistability of acinar and islet cell fates. Inhibition of one or both modes of signaling results in transdifferentiation from the acinar to the islet cell fate, either by dedifferentiation to a multipotent state or by direct lineage switching.

**Conclusions:**

This study provides a theoretical framework to understand the role of contact-mediated signaling in pancreatic cell fate control that may help to improve acinar-to-islet cell transdifferentiation strategies for *β*-cell neogenesis.

## Background

In the course of embryonic development, cells become progressively more specialized. Yet, it is becoming increasingly clear that adult differentiated cells retain the ability to change cell fate under certain conditions [1,2]. Novel approaches in regenerative medicine aim at harnessing this cell type plasticity in order to replace diseased or damaged tissue by targeted conversion of cells from other tissues [3]. Transdifferentiation, also known as lineage conversion, from one cell type to another often involves a dedifferentation step to reinstate multipotency, but it is also possible to force cells to switch lineages directly [4]. Cells can be reprogrammed by ectopic expression of specific transcription factors using viral transduction [5,6]. However, some cell types can also be converted without genetic manipulation, by merely changing the cellular microenvironment. For many purposes, microenvironment-induced conversion may be preferable since it avoids the risks of random viral integration [7]. Contact-mediated signals from neighboring cells constitute a major part of the cellular microenvironment and recent studies have highlighted the importance of cell-cell contacts and surface-bound signals for pluripotency and cell type stability [8-14]. Yet, little is known about the regulatory effects of contact-mediated signals on cell fate stability and cell type conversion. In this paper, we investigate the role of contact-mediated signaling mechanisms in transdifferentiation by a theoretical study of cell fate control in the pancreas.

The pancreas is an organ with dual exocrine/endocrine functions. Acinar cells produce digestive enzymes that enter into the gut, whereas *α* and *β*-cells, organized in the islets of Langerhans, release hormones into the blood stream for glucose homeostasis. Disruption of this homeostasis in diabetic patients is caused by a loss of functional *β*-cells. Conversion of cells from other pancreatic tissues into new *β*-cells has been proposed as a replacement therapy [15]. Acinar cells are interesting candidates as a source for transdifferentiation because of the common developmental origin of exocrine and endocrine cells as well as the abundance of acinar cells in the pancreas [16]. In fact, reprogramming of acinar cells into new *β*-cells has already been demonstrated *in vivo* in mice using ectopic expression of key transcription factors using viral transduction [17]. Intriguingly, such transdifferentiation has also been demonstrated in *in vitro* cultures without genetic manipulation, using only microenvironmental changes [18-21]. These studies show that adult acinar cells spontaneously dedifferentiate upon loss of cell-cell contacts by enzymatic tissue dissociation. Transcription factors and signaling pathways such as Notch signaling are reactivated which normally are only expressed during development. These progenitor-like cells can be converted into *β*-cells, although the yield is typically very low [19-22]. Interestingly, it has been found that the efficiency of lineage conversion can be improved dramatically by inactivation of Notch signaling [23].

These findings suggest that at least two contact-mediated or lateral signaling pathways are involved in acinar-to- *β*-cell conversion. First, dedifferentiation seems to be controlled by the loss of a stabilizing signal that is mediated by contact with adjacent acinar cells and is required for the maintenance of the acinar identity [16]. Second, redifferentiation into the endocrine lineage of islet cells seems to be hampered by contact-mediated Notch signaling [23] in a mechanism known as lateral inhibition, as previously described for pancreas development [24]. Understanding how these lateral signaling pathways act together in regulation of cell type stability and conversion dynamics can be an important step towards the development of non-genetic methods of *β*-cell neogenesis.

In this study, we construct and analyze a mathematical model that combines gene regulation with two contact-mediated signaling mechanisms: lateral inhibition and lateral stabilization. Using a combination of bifurcation analysis and numerical simulation, we find that multistability of gene expression states underlies the potential of acinar-to-islet cell conversion. Whereas loss of lateral stabilization causes a step-wise conversion through a multipotent progenitor-like state, additional loss of lateral inhibition induces the direct transdifferentiation from acinar to islet cells. In addition, cell density as well as the size and structure of cellular aggregates are found to affect the efficiency of conversion. Our results demonstrate that the combination of two lateral signaling mechanisms suffices to reproduce observations of acinar-to-islet cell conversion. By clarifying the role of lateral signals in lineage conversion, this new theoretical framework may contribute to improving strategies of microenvironment- induced transdifferentiation in general and to *β*-cell neogenesis in particular.

## Methods

Gene regulatory networks can be mathematically modeled and analyzed in terms of differential equations [25]. This can help to understand the complex feedback mechanisms underlying cell fate control [26]. By means of model analysis, one can reveal the existence of attractors that represent cellular phenotypes and understand the dynamics between states [27-29]. Using such a systems biological approach, we have previously shown that the results of genetic reprogramming experiments in the pancreas can be predicted from the hierarchical topology of the underlying gene regulatory network [30]. In the present study, we construct a minimal model of the gene regulatory network and contact-mediated signaling pathways underlying endocrine/exocrine cell fate decisions and maintenance in the pancreas and analyse this model using a combination of bifurcation analysis and tissue-scale lattice simulation.

**Figure 1:**
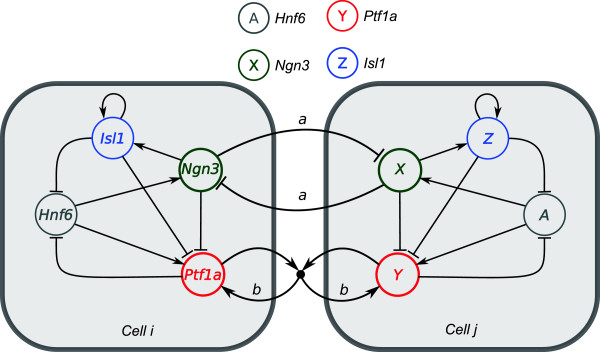
**Gene regulation and lateral signaling network.** In cell *i*, the common names of the transcription factors are used. In cell *j*, these are replaced by the respective model variables. Cells *i* and *j* are coupled by lateral inhibition of factors *X*, and by lateral stabilization between factors *Y*. For each cell, the upstream factor *A* induces expression of *X* and *Y*, while *X* also induces *Z* expression, which activates itself. Both endocrine factors *X* and *Z* antagonize exocrine factor *Y*. Once differentiated, the markers *Y* and *Z* down-regulate *A*. Parameters in small lower case represent strengths of the interactions.

The state of each cell is specified by four variables, *A*, *X*, *Y*, *Z* representing the expression levels of key transcription factors. Whereas *X* and *Y* correspond to core fate-determining genes and are involved in contact-mediated signaling, the factors *A* and *Z* represent up- and downstream factors (see Figure 1). More specifically, the factor *X* represents the pro-endocrine transcription factor *Ngn3* that is transiently expressed during early pancreas development and participates in Notch-mediated lateral inhibition [24,31]. *Ngn3* activates the expression of the membrane-bound Notch ligand *Delta-like1 (Dll1)*[24]. Reversely, activated Notch signaling causes inhibition of *Ngn3* by the transcriptional repressor *Hes1*[32]. As a result, neighboring cells compete for endocrine commitment by mutual inhibition of *Ngn3* expression, in a mechanism called lateral inhibition [33,34]. The factor *Z* represents a terminal endocrine fate marker downstream of *Ngn3* such as *Isl1*[31,35] that, once induced, retains its expression by positive auto-activation. As an islet cell maturation factor, it acts to repress the expression of upstream factor *A*.

The factor *Y* is interpreted as *Ptf1a*, which is the only transcription factor known to be necessary and sufficient to induce the exocrine cell fate [36,37], but is expressed in all pancreatic progenitor cells [38]. Based on experimental evidence that adult acinar cells lose *Ptf1a* expression upon loss of physical cell-cell contact [16,18-21], we assume that factor *Y* is involved in lateral stabilization. Lateral stabilization provides a positive feedback loop between *Y*-expressing neighboring cells [39]. The rate of *Y* production is up-regulated by its simultaneous expression in neighboring cells. Mathematically, this is represented by a multiplication, such that non-*Y*-expressing cells do not participate in lateral stabilization. Although the molecular details of a lateral stabilization pathway are unclear, such conditional activation is, in principle, consistent with both cadherin/*beta*-catenin signaling [13] as well as with *Mist1*-mediated gap junctional communication [14]. In both cases, cells need to express monomeric proteins that form homotypic transmembrane complexes in order to signal to adjacent cells.

Although the endocrine and exocrine markers are mutually exclusive [40], the underlying regulatory mechanisms remain unresolved. One model holds that *Nkx6.1*, a pro-endocrine factor downstream of *Ngn3*[41,42], antagonizes the expression of *Ptf1a*[43]. Independent of the precise molecular pathway, we assume that (pro-) endocrine factors *X* and *Z* independently suppress the expression of *Y* leading to the restriction of the latter factor to the exocrine compartment.

Both *Ngn3* and *Ptf1a* are known to be induced by the upstream factor *Hnf6*, either directly [44] or indirectly [45,46]. To reflect this fact in the model, factor *A* induces the expression of *X* and *Y*. Both *Hnf6* and *Ngn3* are down-regulated during late developmental stages and are not expressed in the adult pancreas under normal circumstances [31]. In the model, this is captured by negative feedback of the terminal islet and acinar markers, *Z* and *Y*, on the inducing factor *A*. Indirectly, this also causes the down-regulation of *X*.

These gene-gene and cell-cell interactions can be formulated in terms of the following system of stochastic differential equations using Hill kinetics (parameters as in Table 1):

(1) $$\begin{array}{ll} \frac{\text{dA}}{\text{dt}} & =\frac{1}{1+rY^{n}+rZ^{n}}-A\; \end{array}$$

**Table 1:** Variables and parameters

	**Symbol**	**Description**	**Value**	
			***Embryo***	***Adult***
**Variables**	*A*	Expression of transcription factor *Hnf6*	1	0
	*X*	Expression of transcription factor *Ngn3*	0	0
	*Y*	Expression of transcription factor *Ptf1a*	0	1
	*Z*	Expression of transcription factor *Isl1*	0	0
	$\bar{X}$	Average *Ngn3* expression in neighboring cells	0	0
	$\bar{Y}$	Average *Ptf1a* expression in neighboring cells	0	1
**Parameters**	*a*	Strength of lateral inhibition *X*⊩⊣*X*	1000	
	*b*	Strength of lateral stabilization *Y*⇔*Y*	2000	
	*c*	Strength of inhibition *X*⊣*Y* and *Z*⊣*Y*	500	
	*q*	Strength of induction *A*→*X* and *A*→*Y*	10^−4^	
	*r*	Strength of inhibition *Y*⊣*A* and *Z*⊣*A*	100	
	*s*	Strength of autoactivation *Z*→*Z*	50	
	*n*	Hill coefficient, nonlinearity of reactions	3	
	*η*_*x*_	Noise amplitude on *X*	10^−3^	
	*η*_*y*_	Noise amplitude on *Y*	10^−3^	

Parameters and initial conditions of the mathematical model (equation 1-4). Parameter values are chosen identical for embryonic and adult conditions, configured with different initial conditions.

(2) $$\begin{array}{ll} \frac{\text{dX}}{\text{dt}} & =\frac{qA^{n}}{q+a{\bar{X}}^{n}}-X+\xi_{x}(t)\; \end{array}$$

(3) $$\begin{array}{ll} \frac{\text{dY}}{\text{dt}} & =\frac{qA^{n}+b{\left(Y\bar{Y}\right)}^{n}}{q+b{\left(Y\bar{Y}\right)}^{n}+cX^{n}+cZ^{n}}-Y+\xi_{y}(t)\; \end{array}$$

(4) $$\begin{array}{ll} \frac{\text{dZ}}{\text{dt}} & =\frac{X^{n}+sZ^{n}}{1+sZ^{n}}-Z\; \end{array}$$

The terms $\bar{X}$ and $\bar{Y}$ denote the average expression of *X* and *Y* in the directly adjacent neighboring cells. To implement lateral inhibition, production of *X* is inhibited by the expression of *X* in neighboring cells, $a{\bar{X}}^{n}$, independent of its own activation. In contrast, the multiplicative term representing lateral stabilization, $b(Y{\bar{Y)}}^{n}$, acts to stabilize a pre-existing expression. This requires the cell-autonomous activity of *Y* in both cells.

The additive stochastic terms *ξ*(*t*), accounting for variability in gene expression or signaling noise, are random variables with a Gaussian white noise distribution *N*(0,*η*) with mean 0 and amplitude *η*. The Hill coefficient *n* is chosen such that the system exhibits non-linear step-like behavior (*n*=3). The model variables are scaled in such a way that the steady state expression of all factors is between 0 and 1. Parameter values are chosen such that the acinar cell fate (cells with *Y*≈1) and islet cell fate (*Z*≈1) are mutually exclusive. For brevity, in the presentation of the results below, *Y*^+^ cells are acinar, *X*^+^ cells are islet progenitors, and *Z*^+^ cells are islet cells.

The states and (in)stabilities of the above model were studied using bifurcation analysis. Numerical simulation of a hexagonal lattice of cells was performed to study the spatiotemporal dynamics at the tissue scale. Analysis and numerical simulation were performed using GRIND (phase plane analysis) [47], XPPAUT (bifurcation analysis) [48] and our modeling environment Morpheus (lattice simulations) [49]. The stochastic differential equations were solved using the 2^*nd*^ order Heun-Maruyama method with time step size *dt*=0.02. The model description for lattice simulations in Morpheus is available as Additional file 1.

## Results

### Multistability of acinar and islet cell fates

Cell fates are characterized by stable patterns of gene expression. Whether a set of interacting genes is able to reach one or more stable states depends on their interaction topology as well as on the strengths of interaction. To investigate the cell fates that can appear in our model, we studied the existence of stable states and their dependence on parameter values for lateral signaling by performing a bifurcation analysis.

**Figure 2:**
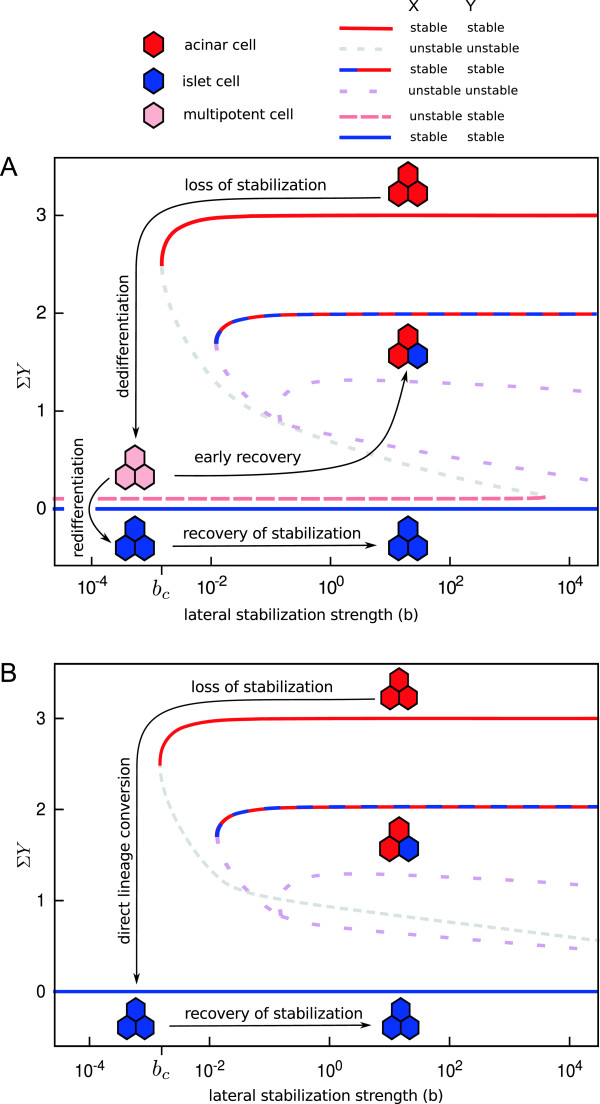
**Bifurcation analysis.** Stability of cell fates change as a function of strength of lateral stabilization. Bifurcation diagram showing stable attractors (solid) and unstable states (dashed) for a minimal tissue consisting of three-cells (hexagons). Arrows indicate trajectories after loss of stabilization. **(A)** In presence of lateral inhibition, *a*>*a*_*c*_, loss of stabilization, *b*<*b*_*c*_, causes dedifferentiation towards a progenitor-like multipotent state. If lateral stabilization is recovered at this early stage, the developmental process is recapitulated and a mixed pattern of both cell fates arises. In contrast, if stabilization remains inhibited, cells redifferentiate into islet cells. **(B)** In absence of lateral inhibition, *a*<*a*_*c*_, loss of stabilization results in direct lineage conversion, due to the absence of a progenitor-like multipotent state. This unstable multipotent state vanishes at *a*_*c*_ in a saddle-node bifurcation with another solution branch of similar *Y*-values but higher *Z* activity which is additionally unstable against perturbations in *Z* and therefore omitted in (A). Note that *ΣY* is a projection of a high (12)-dimensional space, such that intersections do not imply bifurcations or changes in stability as these need not intersect in the actual state space. In the legend, the stability of *X* or *Y* means (un)stable with respect to perturbations in variable *X* or *Y*, respectively. With parameters as in Table 1, *a*_*c*_≈0.0017 and *b*_*c*_≈0.012.

Due to lateral signaling, the fates of individual cells depend on the states of neighboring cells. Therefore, we analyzed a system of three cells representing a minimal tissue that is able to show all possible configurations present in larger systems (the mixed state does not occur for less than 3 cells). To study how the stability of cell fates changes while varying the strength of the lateral stabilization mechanism *b*, we recorded the summed expression level of exocrine factor *Y*. This reduces the high-dimensional state space to a single dimension and provides information on cell fates as well as their spatial pattern. The solid lines in the bifurcation diagram in Figure 2A show that *Y* expression has three stable states over a wide range of parameter values. For these values of *b*, the three cells can have either acinar fates (*Y*=3), islet cell fates (*Y*=0) or have mixed fates (*Y*=2), depending on initial conditions or history of gene expression.

This multistability of acinar and islet cell fates has several interesting consequences. The key observation is that a critical value *b*_*c*_ exists, below which the stable steady state for the acinar fate disappears, while the islet cell fate remains stable. Thus, loss of the stabilizing effect of lateral signaling effectively moves the system towards a region in parameter space where the acinar cell fate no longer exists. Therefore, upon such a change in parameter values, acinar cells lose their exocrine markers and dedifferentiate spontaneously. In the presence of lateral inhibition (Figure 2A) cells adopt a multipotent progenitor-like fate. This state is stable against perturbations in *Y*, but unstable against perturbations in *X*, which implies that noise on *X* can change this state. If lateral stabilization is recovered at this multipotent stage, the system moves towards a steady state with mixed acinar and islet cell fates, recapitulating the cell fate decision and spatial pattern observed during pancreas development [39]. If, however, disruption of lateral stabilization continues, cells differentiate into the islet cell lineage. After completing the lineage conversion, the islet fate is stable in the sense that recovery of lateral stabilization does not reverse conversion.

Interestingly, the bifurcation analysis shows a different behavior in the absence of lateral inhibition (Figure 2B). In this case, multipotent progenitor-like steady state does not exist. This implies that acinar cells cannot dedifferentiate towards a progenitor-like state upon loss of lateral stabilization. Instead, cells undergo direct lineage conversion from the acinar to the islet lineage, rather than passing through a state of multipotency.

In conclusion, bifurcation analysis reveals (1) that lateral stabilization accommodates multistability of the acinar and islet cell states, (2) that transient loss of lateral stabilization can cause the conversion of acinar to islet cells and (3) that concomitant suppression of lateral inhibition leads to direct conversion, bypassing the multipotent progenitor-like state. Yet, bifurcation analysis does not provide insight into the spatiotemporal dynamics for which we next turn to numerical simulations.

### Cell fate decision and patterning during pancreas development

Cells undergoing acinar-to-islet cell conversion transiently express various factors and activate signaling pathways normally only observed during development (*Pdx1*, *Hnf6*, *Ngn3*, *Notch*, *Dll1*) [21,23]. This suggests that at least a part of the developmental regulatory network is reactivated [16] and that cell fate decisions during organogenesis and cell type conversion are governed by the same regulatory mechanisms. Under this assumption, the model proposed here for acinar-to-islet cell conversion is expected to reproduce the cell fate decision between the exocrine or endocrine lineage during embryonic development.

To test whether the proposed model holds for the conditions during embryonic development, we simulated the model using initial conditions that represent the gene expression in early pancreatic progenitor cells. In the mouse, the inductive factors *Hnf6* and *Hnf1β*, that act upstream of lineage-associated factors *Ngn3* and *Ptf1a*, are first detected around E9 [38]. At this stage, *Ngn3* and *Ptf1a* themselves are not yet expressed. Accordingly, the early embryonic state is accounted for in our model by the homogeneous expression of *A* (Table 1).

**Figure 3:**
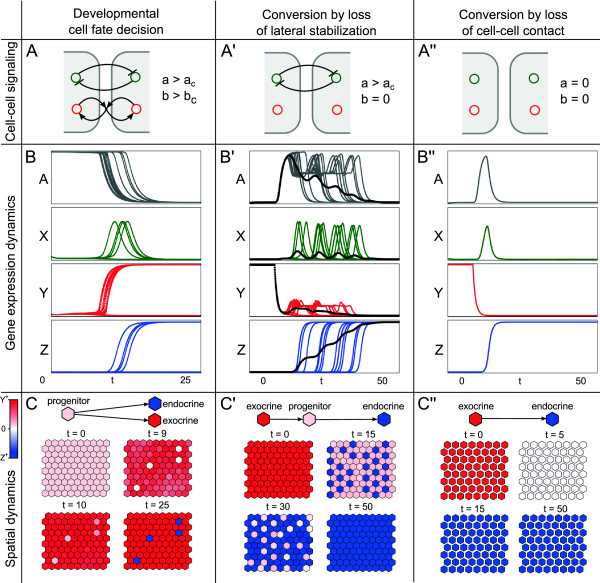
**Dynamics of cell fate control.** Dynamics of cell fate decisions during development (left column) and lineage conversion (middle and right columns). **(A)** Sketch of cell-cell signaling condition. **(B)** Expression of transcription factors over time. A: *Hnf6*, X: *Ngn3*, Y: *Ptf1a*, Z: *Isl1*. Black lines in B’ depict population averages. **(C)** Emergent spatial patterns, representing cell fates by colors. Color coding: *Y*^+^ acinar cells are red, *Z*^+^ islet cells are blue, and *Y*^−^*Z*^−^ cells are white. Initial condition for development is *A*=1,*X*=*Y*=*Z*=0 and for conversion is *Y*=1,*A*=*X*=*Y*=0. Parameters as in Table 1. Movies of the spatiotemporal dynamics are available as Additional files (2, 3 and 4).

Figure 3B shows that during simulation, *A* activates the expression of both *X* and *Y*. For a transient period, these factors are co-expressed in all cells at low or intermediate levels of expression. The “promiscuous” co-expression is typical of multipotent progenitor cells and is also observed in pancreatic progenitors [40]. During this phase, mutual inhibition between cells maintains a low-level expression and thereby suppresses differentiation into either lineage, similar to the role of Notch signaling in pancreatic development known as “suppressive maintenance” [50]. After noise introduces variation in *X* expression between cells, these differences become amplified by lateral inhibition and result in a divergence of *X* expression. Factor *X* activates islet cell differentiation by activating *Z* and is only transiently expressed itself, as is known for *Ngn3*. Reversely, in the *X*^−^ surrounding cells, *Y* is no longer inhibited and is upregulated. Through lateral stabilization, *Y*^+^ cells induce the expression of *Y* in neighboring progenitor cells (with low *Y* expression) which results in wave propagation, in a process traditionally known as homeogenetic induction [51]. Maturation into either lineage results in suppression of upstream factor *A* which leads to the downregulation of the pro-endocrine factor *X*, while *Y* is maintained by lateral stabilization. In line with experimental observations, both factors (*Hnf6* and *Ngn3*) are not expressed after the cell fate decision and in the adult pancreas.

Interestingly, the spatial patterns generated by the model are also in line with reports of the scattered distribution of nascent islet cells in the early pancreatic epithelium [52]. The combination of lateral inhibition (creating an alternating pattern of acinar and islet cells) with lateral stabilization (creating homogeneous fields of acinar cells) results in the establishment of a scattered spatial distribution of endocrine cells in a mainly exocrine tissue (see Figure 3C) [39].

In short, under initial conditions representing early pancreas development, the key features of gene expression and patterning in the developing pancreas are reproduced by the model: (1) promiscuous expression of the lineage-associated factors *Ngn3* and *Ptf1a*, (2) the transient expression of the pro-endocrine factor *Ngn3* and (3) the scattered spatial patterning of committed islet cells.

### Loss of lateral stabilization causes sequential conversion

To understand the dynamics of acinar-to-islet cell conversion upon loss of lateral stabilization, simulated cells were initialized with an acinar-like gene expression profile in which only the exocrine factor *Y* is expressed (see Table 1). The system was initialized with lateral stabilization strength *b*>*b*_*c*_ to ensure the stability of the acinar-like state under these conditions. After a given period, lateral stabilization was lost, *b*=0, marking *t*=0.

As shown in Figure 3B’, the acinar state is stable as long as lateral stabilization strength *b*>*b*_*c*_, representing intact acinar tissue. However, immediately following the loss of lateral stabilization, cells lose the expression of exocrine marker *Y*. The lack of the maturation factor *Y* leads to the re-activation of the upstream factor *A*. Since *A* induces low levels of both *X* and *Y*, at this stage, the expression pattern is identical to the early embryonic situation. Thus, loss of lateral stabilization causes cells to return towards the multipotent progenitor-like cell state. If the absence of lateral stabilization continues, the subsequent dynamics differ from the embryonic cell fate decision discussed above. Specifically, nascent islet cells arise in an alternating spatial pattern as a result of lateral inhibition between *X*^+^ cells (Figure 3C). Yet, this pattern is not stable. After a cell has committed to the islet lineage by transactivating the endocrine marker *Z*, it looses expression of *X*. Therefore, cells adjacent to endocrine *Z*^+^ cells are no longer inhibited and will start to express *X* themselves. As a result, some of the neighboring cells also commit to the endocrine lineage, after which the process is repeated. This step-wise conversion of cells within the tissue results in a complex spatiotemporal patterning process (Figure 3C’). Under these idealized conditions, eventually all cells commit to the islet cell lineage. If, however, lateral stabilization is recovered before cells have redifferentiated, the cell type conversion is arrested which significantly decreases the efficiency of conversion (data not shown). Recovery does not revert newly committed islet cells back to acinar fate, since the islet cell state is stable, independent of lateral stabilization.

These results are in line with *in vitro* experiments showing spontaneous dedifferentiation upon enzymatic disassociation and disruption of cadherin-mediated cell-cell adhesion [13,20-22]. Furthermore, these results suggest that acinar-to-islet cell conversion ensuing loss of lateral stabilization is a relatively slow process due to the fact that lateral inhibition prevents neighboring cells from completing transdifferentiation simultaneously.

### Loss of lateral inhibition accelerates conversion

In the embryo, disruption of the Notch signaling pathway is known to cause precocious endocrine commitment [24]. Moreover, its inhibition in adult acinar cells can dramatically increase the efficiency of acinar-to-islet cell type conversion [23]. Since one of the roles of Notch signaling in the developing pancreas is lateral inhibition, we examined the dynamics of the model after a sudden loss of lateral inhibition. As before, we used the acinar-like initial conditions (Table 1), but now both lateral stabilization and lateral inhibition were lost, *a*=*b*=0, after a given period.

Immediately ensuing this manipulation, *Y* expression rapidly decreases, causing the reactivation of *A* expression, as described before. However, in this case, the dedifferentiated cells do not return to a multipotent state with “promiscuous” co-expression. Instead, all cells simultaneously upregulate the pro-endocrine factor *X* since they are not inhibited by their neighbors (see Figure 3B”). Finally, after the transactivation of *Z* by *X*, the factors *A* and *X* are suppressed again, leading to an adult islet fate in all cells. Compared to the loss of stabilization, the additional loss of lateral inhibition results in a much faster dynamical process of lineage conversion. In line with results obtained *in vitro*[23], our model shows that concomitant inhibition of lateral inhibition accelerates acinar-to-islet conversion. Here, this observation is explained by the fact that, under disruption of lateral inhibition, the unstable steady state representing the multipotent progenitor state does not exist, as predicted by bifurcation analysis (Figure 2B).

Note that disruption of lateral inhibition alone (*a*=0, *b*>*b*_*c*_) does not affect acinar cell stability, since the pro-endocrine factor *X*, which is involved in this feedback between cells, is not expressed in adult acinar cells. Therefore, without loss of lateral stabilization, cells maintain their acinar identity.

### Cell density affects conversion efficiency

**Figure 4:**
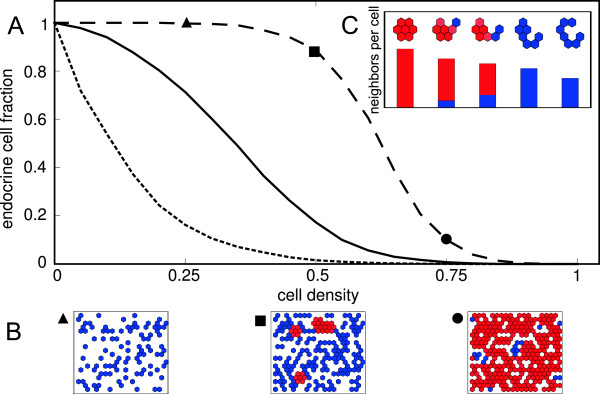
**Cell density affects conversion efficiency.(A)** The fraction of acinar cells that convert to islet cells increases with decreasing cell density, as shown for three values of lateral stabilization strength, *b*=1 (dotted), *b*=0.1 (solid), *b*=0.01 (dashed). **(B)** Examples of the steady-state situation (acinar cells in red and islet cells in blue) for three different cell densities as indicated on the dashed curve (*b*=0.01, densities 0.25, 0.50 and 0.75). Note the presence of compact clusters of stable acinar cells in the middle panel. **(C)** Shape of cellular aggregates determines the efficiency of conversion. A decrease in compactness, measured as average neighbors per cell, increases the islet cell yield. Parameters as in Table 1, *b* as indicated.

If the disruption of contact-mediated signaling influences the efficiency of acinar-to-islet cell conversion, loss of physical contacts between cells could replace molecular manipulation. To study the effect of cell-cell contacts, we performed simulations with varying densities of acinar cells. As expected, it was found that conversion efficiency increases with decreasing cell density (see Figure 4). For extreme cases, the reason behind this is evident. At high densities, most cells have many contacts with neighboring acinar cells and the stabilizing positive feedback prevents their dedifferentiation. Conversely, at low density, most cells are isolated and do not receive stabilizing (or inhibiting) cell-cell signals. Consequently, these cells can complete transdifferentiation. However, for more realistic intermediate cases in which cells are part of small aggregates, the situation becomes nontrivial. Here, the probability of cell conversion depends on both size and shape of the cellular aggregate. Although cells in larger clusters are generally more stable, this stability also depends on the spatial arrangement of cells in the aggregate (Figure 4C). Because the dedifferentiation of one cell weakens the stability of its neighboring cells, waves of dedifferentiation and conversion can propagate through the aggregate, depending on the average number of neighboring cells that reflects both density and configuration of a cell cluster.

These results show that, in the context of our model, the size and the structure of cellular aggregates affects the efficiency of lineage conversion. This implies that the degree of dissociation of acini by enzymatic digestion is predicted to have large impact on islet cell yield. More generally, the use of low cell densities or, alternatively, inhibition of reaggregation of cells, is predicted to increase the efficiency of acinar-to-islet cell conversion *in vitro*.

## Discussion and conclusion

Forcing adult cells to change lineage by altering the microenvironment offers an alternative to the more risky method of virus-mediated nuclear reprogramming [6,7]. Apart from identifying of specific growth factors and small molecules that induce a particular lineage conversion *in vitro*, recent work in this direction also demonstrates that contact-mediated lateral signals are key regulators of cell fate maintenance and multipotency [8-14]. For instance, it was found that loss of cell-cell adhesion between adult acinar cells of the pancreas causes dedifferentiation and enables their conversion into islet cells [13,20]. Together with more recent data showing that inhibition of contact-mediated Notch signaling between these cells significantly improves conversion efficiency [23], this demonstrates that lateral signals are important regulators of cell fate control in the pancreas. However, despite the identification of a myriad of transcription factors and signaling molecules involved in lineage conversion, a coherent understanding of the roles of contact-mediated lateral signals in this process is lacking.

A systems biological approach can help to make sense of complex dynamic regulatory networks through the use of mathematical models and dynamical system theory [25,26]. In previous work, we have adopted this approach to construct a hierarchical multi-attractor model of the pancreatic transcriptional network to understand and propose nuclear reprogramming strategies [30]. In the present study, instead, we have focused on the role of contact-mediated signals on conversion dynamics to predict the outcomes of microenvironment-induced strategies for transdifferentiation.

We have presented a mathematical model that combines gene regulation and lateral signaling in pancreatic cells. We have demonstrated that the crosstalk of two contact-mediated signaling mechanisms (lateral inhibition and lateral stabilization) causes multistability in which both acinar and islet cell fates are stable. Our discovery of the multistable state explains why conversion of acinar to islet cells is possible, even without genetic manipulation. Inhibition of lateral stabilization destabilizes of acinar cells and causes the dedifferentiation of acinar cells towards a progenitor-like multipotent state and invokes the subsequent step-wise conversion towards an islet cell fate. Moreover, we have shown that additional loss of lateral inhibition accelerates the conversion dynamics because, under these conditions, cells undergo a direct lineage switching, without passing through a multipotent state.

Altogether, our results provide a theoretical background to understand studies of acinar-to-islet cell conversion *in vitro*[13,18-20,23]. Moreover, this study offers several testable predictions, such as the impact of cell density, that may be used to improve the efficiency of microenvironment-induced conversion strategies. More generally, our results demonstrate that the crosstalk of multiple lateral signaling mechanisms can generate counterintuitive effects controlling cell fate stability as well as spatial patterning, which deserve further investigation. Furthermore, this study underscores that the identity of cells depends on the multicellular context of the tissue. Therefore, considering the feedback from the tissue level to the genetic level is important in order to understand how cell fate stability and plasticity are controlled.

## Competing interests

The authors declare that they have no competing interests.

## Authors’ contributions

WdB conceived the study, constructed the mathematical model, participated in its analysis and drafted the manuscript. RZ participated in modeling and performed bifurcation analysis and numerical simulation and helped to draft the manuscript. LB participated in the design of the study as well as its coordination and helped in analysis and drafting the manuscript. All authors read and approved the final manuscript.

## Supplementary Material

Additional file 1 **Morpheus XML model description file.** XML file to run lattice simulations in the modeling environment Morpheus [49], which can be downloaded at http://imc.zih.tu-dresden.de/wiki/morpheus. The model is configured to reproduce the simulations of conversion by loss of lateral stabilization (Figure 3B’ and 3C’).
Click here for file

Additional file 2 **Spatial and temporal dynamics: Development.** Movie showing spatial and temporal dynamics during development, as in
Figure 3B and 3C. Left panels show a spatial dynamics in lattice simulation with colors indicating expression levels of *A*, *X*, *Y* and *Z*. Right panels show corresponding gene expression of the same factors over time. Lattice simulation performed with our own modeling software Morpheus [49].
Click here for file

Additional file 3 **Spatial and temporal dynamics: Conversion by loss of lateral stabilization.** Movie showing spatial and temporal dynamics during adult lineage conversion, as in Figure 3B’ and 3C’. Left panels show a spatial dynamics in lattice simulation with colors indicating expression levels of *A*, *X*, *Y* and *Z*. Right panels show corresponding gene expression of the same factors over time. Lattice simulation performed with our own modeling software Morpheus [49].
Click here for file

Additional file 4 **Spatial and temporal dynamics: Conversion by loss of lateral signaling.** Movie showing spatial and temporal dynamics during adult lineage conversion, as in Figure 3B” and 3C”. Left panels show a spatial dynamics in lattice simulation with colors indicating expression levels of *A*, *X*, *Y* and *Z*. Right panels show corresponding gene expression of the same factors over time. Lattice simulation performed with our own modeling software Morpheus [49].
Click here for file
